# Human Olfactory Mesenchymal Stem Cells Are a Novel Candidate for Neurological Autoimmune Disease

**DOI:** 10.3389/fphar.2021.770884

**Published:** 2021-12-10

**Authors:** Chongjun Xiao, Di Lu, Jinshuo Chen, Xiaoyan Chen, Huizhu Lin, Mudan Huang, Shimei Cheng, Yuge Wang, Qiuli Liu, Haiqing Zheng

**Affiliations:** ^1^ Department of Rehabilitation Medicine, The Third Affiliated Hospital of Sun Yat-Sen University, Guangzhou, China; ^2^ The Biotherapy Center, The Third Affiliated Hospital of Sun Yat-Sen University, Guangzhou, China; ^3^ Department of Neurology, The Third Affiliated Hospital of Sun Yat-Sen University, Guangzhou, China

**Keywords:** experimental autoimmune encephalomyelitis, olfactory mucosa–derived mesenchymal stem cells, immunomodulation, mesenchymal stem cell therapy, neuroinflammation

## Abstract

**Background:** Human olfactory mesenchymal stem cells (OMSC) have become a novel therapeutic option for immune disorder or demyelinating disease due to their immunomodulatory and regenerative potentials. However, the immunomodulatory effects of OMSC still need to be elucidated, and comparisons of the effects of different MSCs are also required in order to select an optimal cell source for further applications.

**Results:** In animal experiments, we found neural functional recovery and delayed EAE attack in the OMSC treatment group. Compared with umbilical cord–derived mesenchymal stem cells (UMSC) treatment group and the control group, the OMSC treatment group had a better neurological improvement, lower serum levels of IFN-γ, and a lower proportion of CD4+IFN-γ+ T splenic lymphocyte. We also observed OMSC effectively suppressed CD4+IFN-γ+ T cell proportion *in vitro* when co-cultured with human peripheral blood–derived lymphocytes. The OMSC-mediated immunosuppressive effect on human CD4+IFN-γ+ T cells was attenuated by blocking cyclooxygenase activity.

**Conclusion:** Our results suggest that OMSC treatment delayed the onset and promoted the neural functional recovery in the EAE mouse model possibly by suppressing CD4+IFN-γ+ T cells. OMSC transplantation might become an alternative therapeutic option for neurological autoimmune disease.

## Introduction

In recent years, mesenchymal stem cell (MSC)–based therapy has become a new clinical approach for treating various diseases, but the specific mechanisms of action are still not fully elucidated. Previous studies have demonstrated that different sources of MSCs can improve the neurological function of various animal models through immunomodulation (Bai et al., 2009; Peron et al., 2012; Payne et al., 2013; Donders et al., 2015; Bravo et al., 2016; Shu et al., 2018; Ahmadvand Koohsari et al., 2021). However, the effects of MSCs differ in terms of the tissue source. Payne et al. compared the therapeutic effects of human bone marrow–derived MSC (BMSC), adipose-derived MSC, and umbilical cord–derived MSC (UMSC) and found that the immunosuppressive effects among these MSCs were different (Payne et al., 2013). Thus, these findings suggested that seeking the most appropriate MSC was of great significance.

The human olfactory mucosa is a tissue that promotes neurogenesis throughout life (Johnstone et al., 2015), and its cell components play an important role in repairing damaged olfactory nerves (Schwob, 2002). Olfactory mucosa–derived mesenchymal stem cells (OMSC) lay in the lamina propria of the olfactory mucosa (Lindsay et al., 2020). They present immunoregulatory and regenerative effects in animal experiments, which indicates that they are potential candidates for treating related diseases (Lindsay et al., 2013; Khankan et al., 2016; Rui et al., 2016). Recent research showed that OMSC not only shared common characteristics with other MSC (Lindsay et al., 2013; Rui et al., 2016), such as self-renewal, multi-differentiation, and immunoregulatory potential, but also had extra advantages in promoting neuronal affinity (Veron et al., 2018) and myelin regeneration (Lindsay et al., 2017). Lindsay et al. showed that human OMSC promoted the neural functional recovery *via* enhancing immune regulation and myelin repair in mice with spinal cord injury (Lindsay et al., 2016; Lindsay et al., 2017). For an *in vitro* study, Di Trapani et al. had demonstrated that OMSC had immunomodulatory effects on T cells, but its molecular mechanisms remain unknown (Di Trapani et al., 2013). Thus, the specific mechanisms of OMSC still need to be further elucidated.

In order to study the effect and immunoregulatory mechanism of OMSC, we established an experimental autoimmune encephalomyelitis (EAE) mice model and a co-culture system of human lymphocytes and OMSC. The EAE model is an autoimmune disease model mediated by CD4+ T helper (Th) cells, characterized by the local infiltration of T lymphocytes in the CNS, and recognized as a classical animal model to study the autoimmune disease (Gerdoni et al., 2007; Robinson et al., 2014). A previous study had demonstrated that UMSC ameliorated EAE by regulating the inflammatory response (Liu et al., 2013). Thus, in this study, we aimed to compare the therapeutic effects between UMSC and OMSC to select the potential seed cells and demonstrate the immunomodulatory effects of OMSC.

## Results

### Characteristics of OMSC and UMSC

**FIGURE 1:**
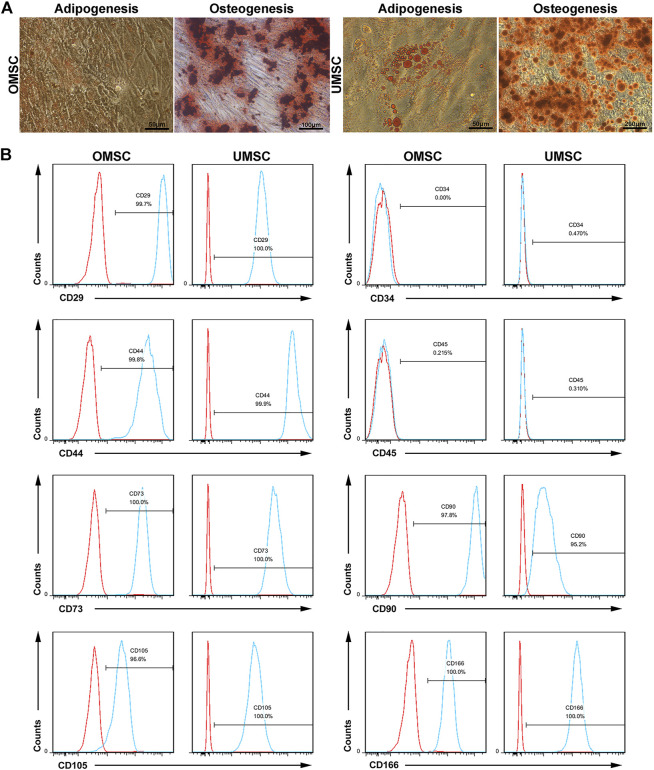
Differentiation potential and phenotypes of the OMSC and UMSC: **(A)** MSCs captured by the microscope. Alizarin Red S chelates with calcium ions to form a red or purplish red complex, which represents the osteogenic formation of OMSC and UMSC. Oil Red O colorizes the lipid inside the MSCs and appears as red lipid droplets, which represents adipogenic formation of OMSC and UMSC. **(B)** Phenotypes of OMSC, including CD29, CD34, CD45, CD44, CD73, CD90, CD105, and CD166, were detected by flow cytometry.

OMSC and UMSC isolated from the human olfactory mucosa and human umbilical cord appeared as spindle-shaped cells and presented adipogenic and osteogenic differentiation potential as determined by Oil Red O and Alizarin Red S staining, respectively (Figure 1A). Phenotype analysis by flow cytometry showed that OMSC expressed CD29, CD44, CD73, CD90, CD105, and CD166 but did not express CD34 and CD45 (Figure 1B).

### OMSC Promotes Neural Functional Recovery *in vivo*

**FIGURE 2:**
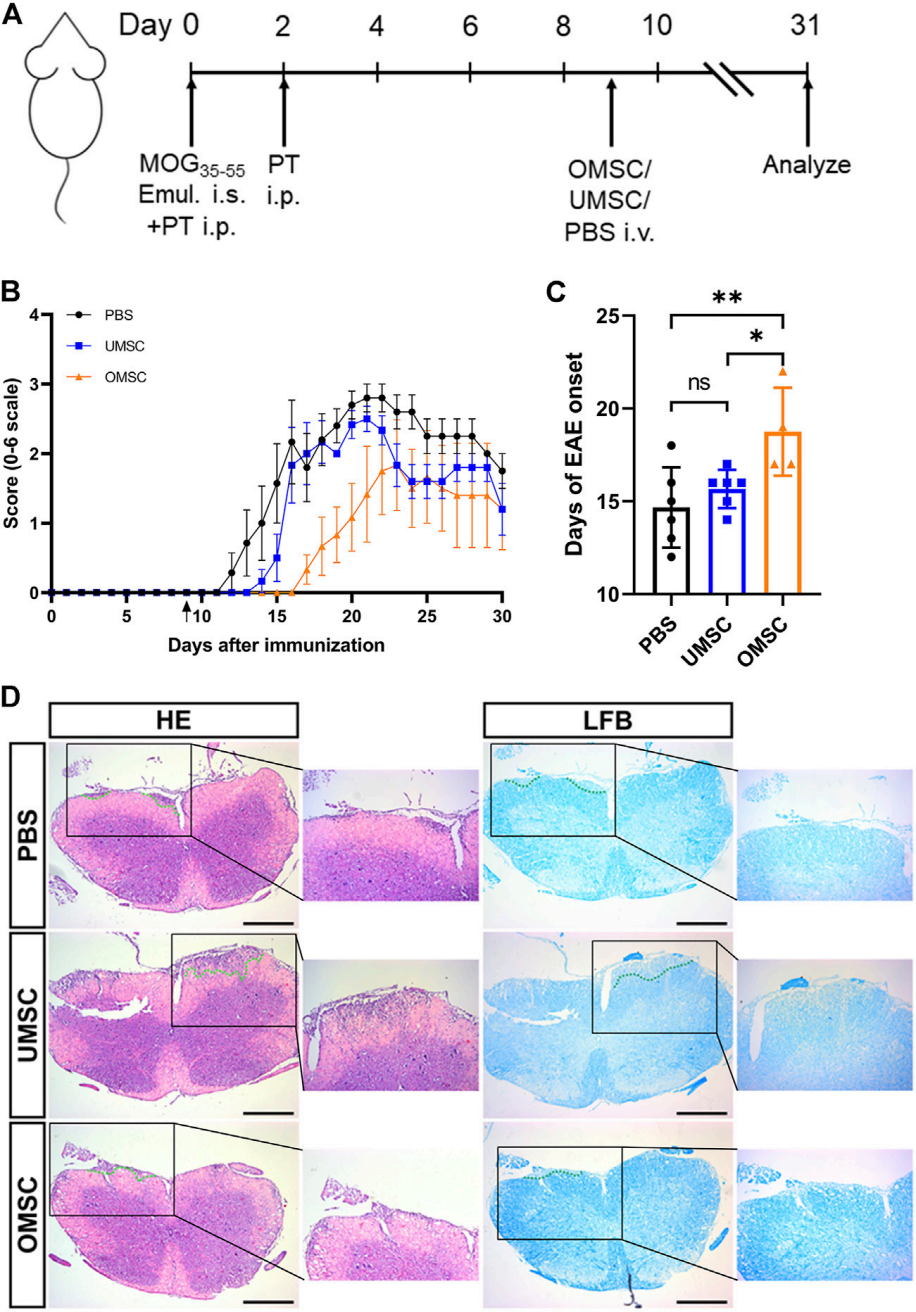
Preventive effects of early intervention of OMSC and UMSC treatments on EAE mice: **(A)** Model diagram of the experimental process. **(B)** Effects of preventive treatment of OMSC and UMSC on the neurological function of EAE mice (*n* = 6–7). **(C)** Effects of preventive treatment of OMSC and UMSC on the onset of EAE in mice. **(D)** HE staining and LFB staining of the spinal cord of EAE mice. i.s.: subcutaneous injection; i.p.: intraperitoneal injection; i.v.: intravenous injection. Bar: 400 μm *p < 0.05; ns: the difference was not statistically significant.

To check the therapeutic effect of OMSC and UMSC on EAE mice, their neurological function was evaluated daily, as shown in Figure 2A. During 31 days of EAE process, the neurological function of the mice treated with OMSC was significantly improved (*p*_OMSC-UMSC_ = 0.001,*p*_OMSC-PBS_<0.001, Figure 2B). On days 16 to 20, daily clinical score differences among the OMSC, UMSC, and PBS treatment groups were statistically significant, showing the extraordinary therapeutic effects of OMSC. The day of EAE onset in each group was statistically significant (Figure 2C, *F* = 5.942, *p* = 0.015). The onset of the OMSC treatment group was later than that of UMSC treatment and control groups (Figure 2C; Table 1, *p*_OMSC-PBS_ = 0.005, *p*_OMSC-PBS_ = 0.024). The incidence within 30 days in OMSC treatment, UMSC treatment, and control groups were 66.7, 100, and 100%, respectively (Table 1), and the mortality were 16.67, 16.67, and 42.86% (Table 1), respectively. In conclusion, EAE mice had improved neurological function, delayed onset time, and reduced incidence and mortality rate after OMSC intervention.

**TABLE 1:** Clinical characteristic of EAE mice treated with MSCs.

	OMSC	UMSC	PBS
Incidence (%)	4/6	6/6	7/7
	66.7%	100%	100%
Mortality (%)	1/6	1/6	3/7
	16.67%	16.67%	42.86%
Mean onset day (d)	18.75 ± 1.18	15.67 ± 0.42	14.67 ± 0.88
(Interval)	(14 ∼ 21)	(14 ∼ 17)	(12 ∼ 18)
Average score	0.58 ± 0.12	0.94 ± 0.18	1.24 ± 0.20

Spinal cord sections of mice in OMSC, UMSC, and PBS treatment groups were stained with HE and LFB (Figure 2D), respectively. It was found that compared with PBS and UMSC groups, the inflammatory and demyelinating areas of the OMSC group showed a downward trend. However, there was no statistical significance in the histological inflammation score and demyelination score among OMSC, UMSC treatment groups, and control group (data not shown).

### OMSC and UMSC Reduced the Serum Level of IFN-γ *in vivo*

**FIGURE 3:**
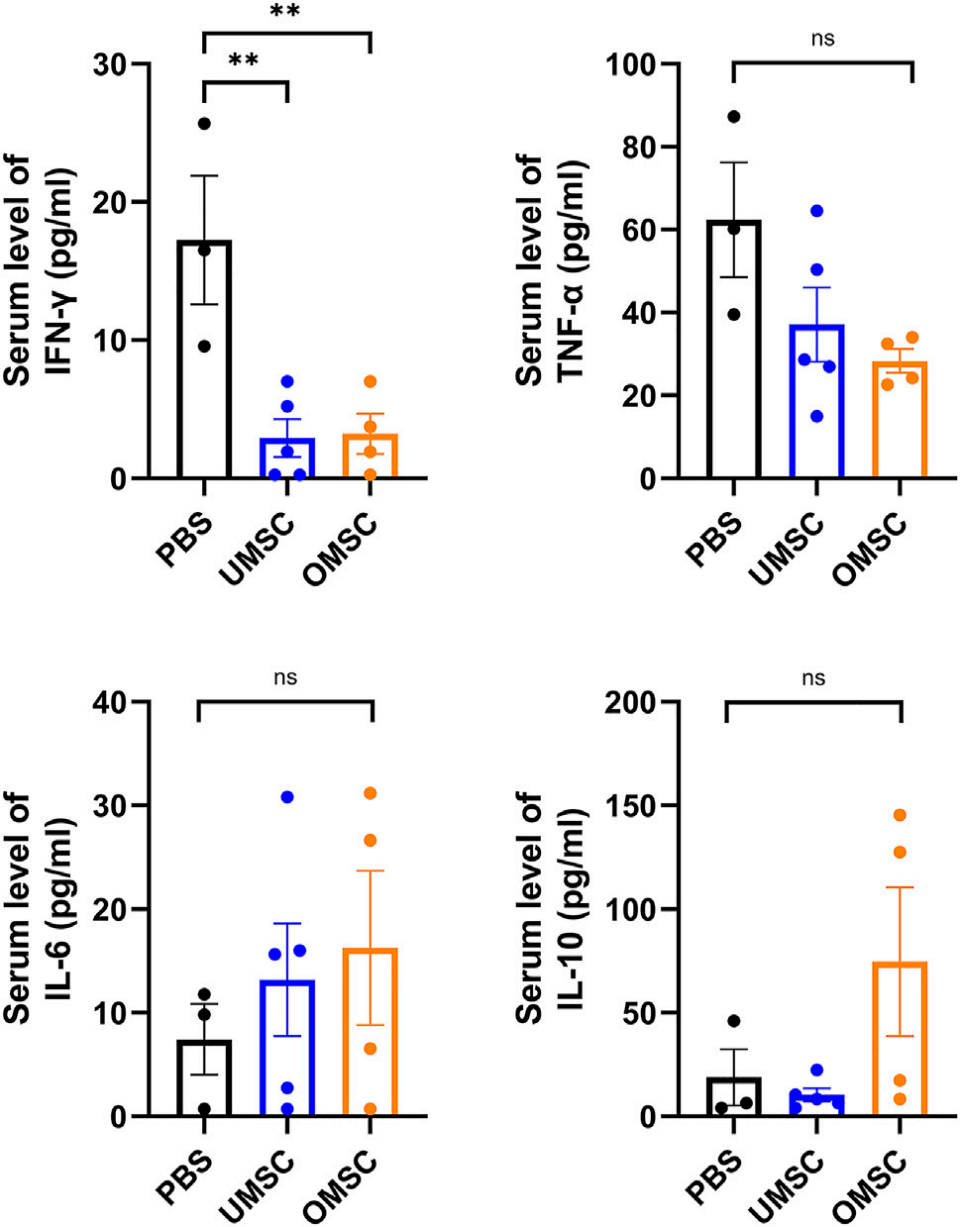
Effects of early intervention of OMSC and UMSC treatments on serum inflammatory cytokines in EAE mice: serum IFN-γ, TNF-α, IL-10, and IL-6 levels in the OMSC treatment group (*n* = 4), UMSC treatment group (*n* = 5), and PBS group (*n* = 3) of EAE mice. *p < 0.05, **p < 0.01, ns: the difference was not statistically significant.

In order to study the effect of OMSC and UMSC on serum inflammatory factors in EAE mice, serum IFN-γ, TNF-α, IL-6, and IL-10 levels were detected by CBA. Differences of serum IFN-γ levels between OMSC treatment, UMSC treatment, and control groups were statistically significant (Figure 3, *p*_IFN-γ_ = 0.004). The serum levels of IFN-γ in UMSC treatment and OMSC treatment groups were lower than those in the control group (Figure 3, *p*_UMSC-PBS_ = 0.002, *p*_OMSC-PBS_ = 0.003). Serum levels of TNF-*α*, IL-10, and IL-6 in the three groups were not statistically significant (Figure 3, *p*_IL-10_ = 0.120, *p*_TNF-α_ = 0.085, *p*_IL6_ = 0.646).

### OMSC Suppressed CD4+IFN-γ+ T Cells *in vitro* and *in vivo*

**FIGURE 4:**
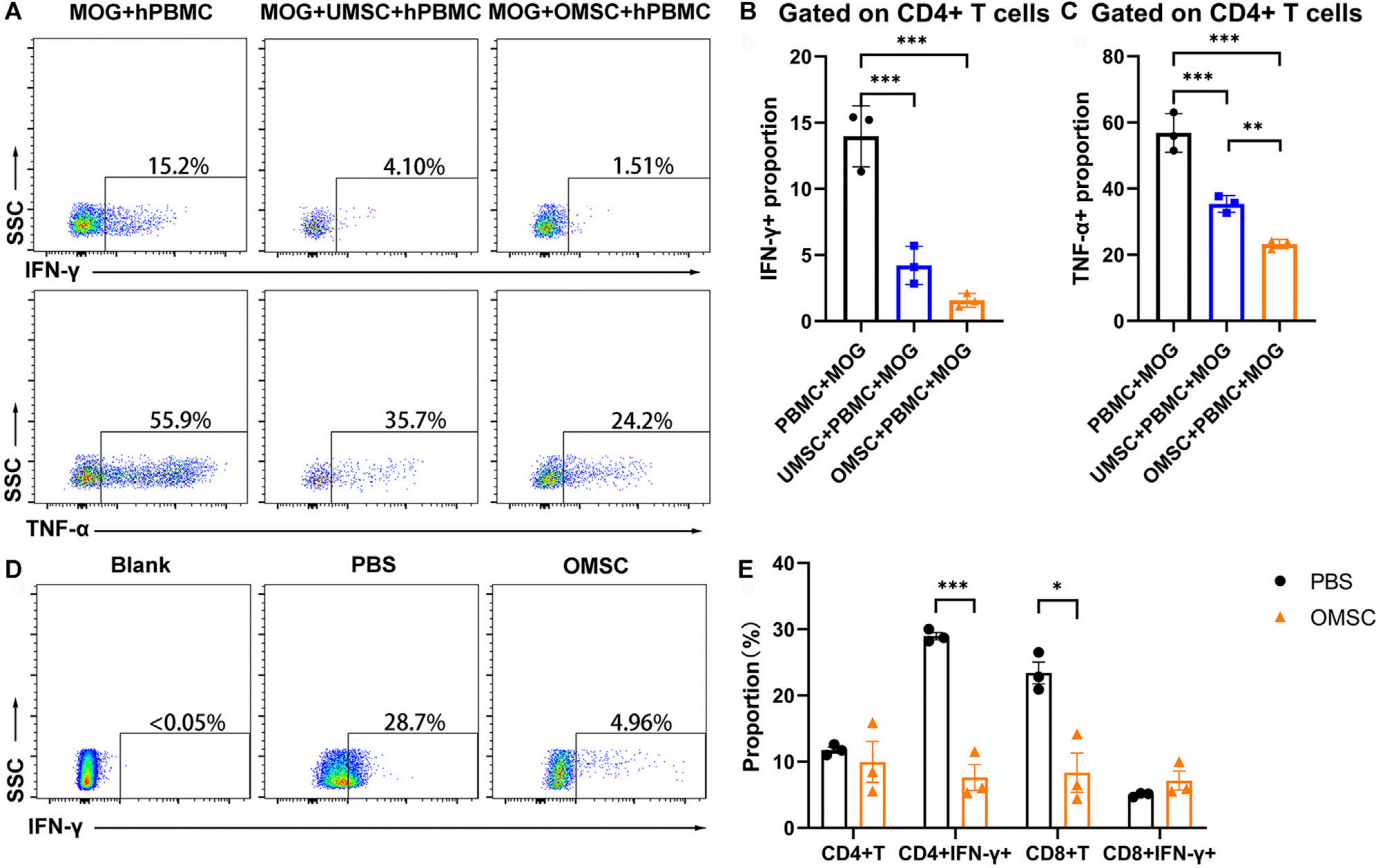
OMSC treatment reduced CD4+IFN-γ+ T lymphocytes: **(A)** Proportion of CD4+IFN-γ+ T cells (Th1) and CD4+TNF-α+ T cells in human lymphocyte single culture, UMSC, and OMSC co-culture groups. **(B, C)** CD4+IFN-γ+ T cells and CD4+TNF-α+ T cells of the co-culture system. **p < 0.01 and ***p < 0.001. **(D)** Proportion of CD4+IFN-γ+ T cells in mice splenic lymphocytes in the OMSC treatment group and PBS group. **(E)** CD4+T cells and CD8+T cells and their IFN-γ expression by splenic lymphocytes in the mice of the OMSC treatment group and PBS group (*n* = 3). *p < 0.05 and ***p < 0.001.

We have found that OMSC better improved neurological function and reduced the serum IFN-γ level in EAE mice compared with UMSC. Since IFN-γ is a crucial cytokine secreted by Th1 lymphocytes, and Th1 lymphocytes, characterized as CD3+CD4+IFN-γ+ cells, play a pivotal role in the development of EAE; we assumed that early preventive treatment of OMSC could downregulate the proportion of CD4+IFN-γ+ T cells. We co-cultured UMSC, OMSC, and human peripheral blood mononuclear cells (PBMCs) and stimulated them with MOG_35-55_ for 48 h to observe the immunomodulatory effects of UMSC and OMSC under MOG_35-55_ stimulation. Since serum TNF-α showed a decreasing trend in EAE mice receiving OMSC treatment, and TNF-α is also an important marker for EAE, CD4+IFN-γ+ T cell proportion and CD4+TNF-α+ T cell proportion were all detected by flow cytometry (Figure 4A). The proportion of CD4+IFN-γ+ T cells (Figure 4B) and CD4+TNF-α+ T cells (Figure 4C) significantly decreased in MOG_35-55_-stimulated PBMC co-cultured with UMSC and OMSC compared to MOG_35-55_-stimulated PBMC cultured alone. Meanwhile, OMSC exhibited a stronger inhibitory effect on CD4+TNF-α+ T cells than UMSC (Figure 4C).

For the *in vivo* study, the mice underwent OMSC preventive treatment, and the control group was sacrificed at the day of EAE onset, and splenic lymphocyte analysis was performed. The proportion of CD4+IFN-γ+ T cells (Th1 cells) in the PBS and OMSC treatment groups was analyzed (Figure 4D). The results showed that the percentages of CD4+IFN-γ+ T cells in the OMSC treatment and PBS groups were 7.61 ± 1.96 % and 28.97 ± 0.54 %, respectively, and the difference was statistically significant (Figure 4E, *t* = 10.51, *p* < 0.001). Interestingly, although EAE induced by MOG_35-55_ is mainly CD4+ T cell–dependent, the proportions of CD8+T lymphocytes were also lower in the OMSC treatment group, indicating a multi-immunomodulatory potential of the OMSC. There was no significant difference in the proportions of CD4+T lymphocytes and CD8^+^ IFN-γ+ T lymphocytes between the two groups. The above mentioned studies highlighted the immunomodulatory effects of OMSCs.

### OMSC Inhibited Human CD4+IFN-γ+ T Cells *in vitro* Partially *via* COX2 Activity

**FIGURE 5:**
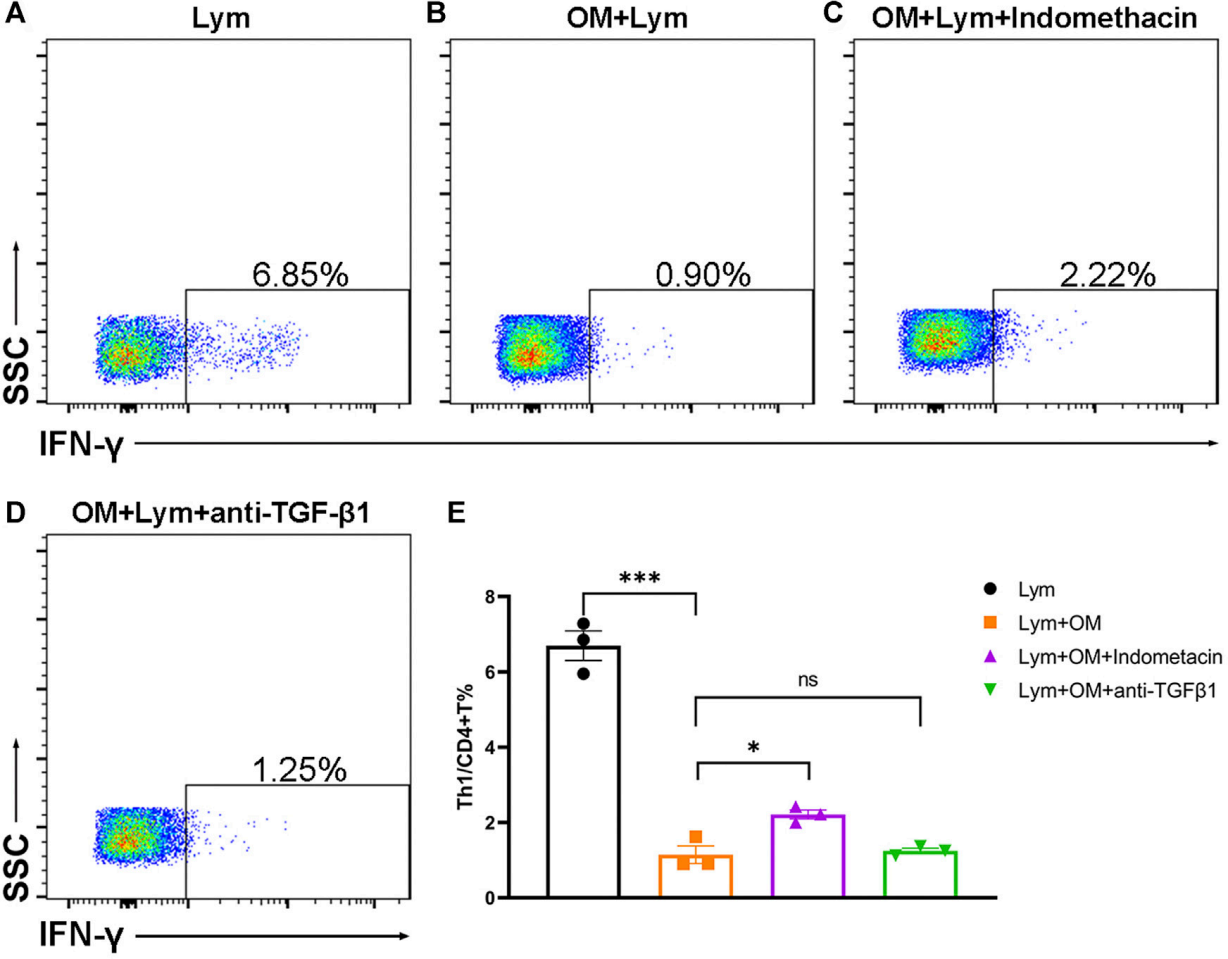
OMSC inhibits the CD4+IFN-γ+ T lymphocyte *in vitro*: **(A–D)** CD4+IFN-γ+ T cell ratio in the human lymphocyte single culture group **(A)**, OMSC co-culture group **(B)**, COX inhibitor group **(C)**, and TGF-β1 inhibitor group **(D)**. **(E)** Difference of CD4+IFN-γ+ T cell ratio among the lymphocyte single culture group, OMSC co-culture group, COX inhibitor group, and TGF-β1 inhibitor group (*n* = 3). Lym: lymphocytes; OM: OMSC. *p < 0.05; ***p < 0.001; ns: the difference was not statistically significant.

In order to study whether OMSC can regulate the T-cell subsets of human lymphocytes and their mechanism, we constructed a co-culture system of OMSC and human PBMC and detected the proportion of CD4+IFN-γ+ T cells by flow cytometry (Figure 5). The results showed that after 48 h of co-culture, the proportion of CD4+IFN-γ+ T cells in the single culture group (Figure 5A) was 6.69 ± 0.39 % and that in the co-culture group (Figure 5B) was 1.14 ± 0.24 %. There was a significant difference in the proportion of CD4+IFN-γ+ T cells between the two groups (Figure 5E, *F* = 119.60, *p* < 0.0001), which confirmed the immunomodulatory effects of OMSC.

Besides, we used indomethacin and TGF-β1 inhibitors to elucidate the possible mechanism of OMSC that inhibited human CD4+IFN-γ+ T lymphocytes. Flow cytometry (Figures 5A–E) was used to detect the proportion of CD4+IFN-γ+ T cells in different groups. The results showed that the percentage of CD4+IFN-γ+ T cells in the COX inhibitor group (indomethacin) was 2.21 ± 0.12 % (Figure 5C) and that in the TGF-β1 inhibitor group was 1.25 ± 0.07 % (Figure 5D), respectively. The ratio of CD4+IFN-γ+ T cells in the single culture group was significantly higher than that in the OMSC co-culture group (Figure 5E, *p* < 0.001); the ratio of CD4+IFN-γ+ T cells in the COX inhibitor group was higher than that in the OMSC co-culture group (Figure 5E, *p* = 0.014). TGF-β1 inhibition has no obvious effect on OMSC-mediated inhibition of CD4+IFN-γ+ T cells. Taken together, these results suggest that OMSCs decreased the proportion of CD4+IFN-γ+ T cells *in vitro* partially *via* COX activity.

## Discussion

In our study, OMSC treatment before the EAE onset effectively increased the neurological improvement in EAE mice compared with PBS and UMSC, followed by a reduced serum level of IFN-γ. We also found that OMSC effectively suppressed CD4+IFN-γ+ T cell proportion *in vivo* and *in vitro*, and this effect is closely related to the COX pathway.

OMSC are a potential candidate for clinical application for their abilities in immunoregulation. At present, there are more than 10 clinical research reports on the olfactory mucosa and its cellular components, which preliminarily confirmed the safety of olfactory mucosa component application (Mackay-Sim et al., 2008) and the potential therapeutic effect (Wu et al., 2012; Chen et al., 2014). Andrews et al. (2016) evaluated the safety of the transnasal endoscopy olfactory mucosa sampling, suggesting that nasal endoscopic extraction of the olfactory mucosa does not affect nasal function and olfaction (IIa evidence). Therefore, autologous transplantation of OMSC is feasible, and it is necessary to further study the efficacy and mechanism of OMSC.

Various studies have shown that OMSC achieve therapeutic role by promoting immune regulation: Lindsay et al. found that the therapeutic effect of OMSC on demyelinating disease might be better than that of BMSC (Lindsay and Barnett, 2017). Meanwhile, Rui et al. (2016) reported that OMSC inhibited the proportion of Th1 cells and IFN-γ secretion in the spleen and suppressed autoimmune arthritis of mice. In addition, Lindsay et al. found that OMSC co-cultured *in vitro* promoted the polarization of SD rat microglia to an anti-inflammatory phenotype (Lindsay et al., 2016). As mentioned before, OMSC may have a better therapeutic effect on immune-related diseases.

We used human OMSC and UMSC for preventive treatment on EAE mice at day 9 and found that the neurological function of mice in the OMSC preventive treatment group was better than that in the UMSC preventive treatment and control groups. The neurological function of the UMSC preventive treatment group showed a certain improvement trend compared with that of the control group, which was consistent with the previous research. In addition, compared with the control group, the mortality of the two MSC preventive treatment groups was lower, and the OMSC prevention group had the lowest incidence (66.7%) among the three groups within 30 days. We also found that the preventive treatment of OMSC and UMSC effectively delayed the onset of EAE in mice. These results also confirmed that the preventive treatment of OMSC improved the neurological function of mice and prevented the occurrence of EAE in mice.

Bai et al. found that stem cells played a therapeutic role mainly through a paracrine secretion–related immune regulation, rather than self-regeneration (Bai et al., 2012). Th1 lymphocytes, which are known as CD4+IFN-γ+ T cells and characterized by the secretion of IFN-γ, have been proven to be closely linked to the pathogenesis of EAE (Cummings et al., 2018). Li et al. found that MSCs could promote the recovery of neural function in EAE mice by increasing the proportion and function of CD5+IL-10 + B cells (Li et al., 2019). Other studies have shown that Th17 lymphocytes were also one of the main inflammatory cells involved in the pathogenesis of MS and EAE mice (Waisman et al., 2015; Liu et al., 2019), and the secretion of IL-17 could promote astrocytes to produce IL-6 and aggravate the inflammatory infiltration of EAE mice (Shan et al., 2017). Some researchers have proposed that MSCs might inhibit the proliferation of peripheral pathogenic T lymphocytes or Th1 lymphocytes (Gerdoni et al., 2007; Bai et al., 2009). These results suggest that inflammatory factors such as IFN-γ, TNF-α, IL-6, and IL-10 may participate in the recovery progress of the neural function in EAE mice mediated by MSCs. In our study, serum levels of IFN-γ, TNF-α, IL-17, IL-6, and IL-10 in OMSC preventive treatment, UMSC preventive treatment group, and control groups were analyzed. However, the serum level of IL-17 in all groups could not be detected accurately using CBA due to its low concentration. It was found that the serum IFN-γ level of EAE mice was affected by OMSC or UMSC preventive treatment. The levels of serum IFN-γ in the OMSC and UMSC preventive treatment groups were lower than those in the control group. There were no significant differences in the serum levels of TNF-α, IL-6, and IL-10 among these three groups, but the serum TNF-α level of OMSC and UMSC showed a certain downward trend (*p*_TNF-α_ = 0.089). Combined with previous studies and our results, OMSC preventive therapy may improve the neurological function of EAE mice by suppressing CD4+IFN-γ+ T cells and its IFN-γ secretion.

Recent studies have shown that transforming growth factor-β1 (TGF-β1) is an important factor acting on the immunosuppressive function of MSCs (Wu et al., 2020). The transforming growth factor-β (TGF-β) family has extensive and diverse effects on metazoan cells and plays a crucial role in regulating immune responses (Liénart et al., 2018). Among family members such as TGF-β1, TGF-β2, and TGF-β3 isoforms, TGF-β1 is the main subtype secreted by immune cells (Travis and Sheppard, 2014).

Di Trapani et al. demonstrated that the immunosuppressive effect of other stem cells (leptomeningeal-derived stem cells) on T cells was also related to the cyclooxygenase (COX) pathway (Di Trapani et al., 2013). The arachidonic acid pathway is a core pathway in the process of human immune regulation. Cyclooxygenase (COX) catalyzes the conversion of arachidonic acid to prostaglandin (PG), standing as the key enzyme in the reaction (Mahboubi Rabbani and Zarghi, 2019). It was believed that PG was mainly involved in the regulation of acute inflammation, but some studies have shown that PG aggravates the progress of arthritis and inflammatory bowel disease and also participates in the occurrence and development of chronic inflammation (Wang and DuBois, 2018; Yao and Narumiya, 2019). Besides, Tonby et al. demonstrated that COX inhibitors effectively inhibited the activation of Th1 cells and reduced inflammatory response (Tonby et al., 2016). At present, the mechanism of OMSC regulating human lymphocytes remains unclear. We assumed that the suppressive effect of OMSC on human CD4+IFN-γ+ T cells (Th1 cells) might be related to TGF-β1 or COX.

In order to elucidate the mechanism of the immunosuppressive effect of OMSCs on CD4+IFN-γ+ T cells, we used indomethacin, a COX inhibitor, or TGF-β1 inhibitor to intervene the OMSC and lymphocyte co-culture system, respectively. After COX inhibition, the proportion of CD4+IFN-γ+ T cells in the COX inhibitor group and co-culture group was statistically different, which indicated that the COX pathway was involved in the inhibitory effect of OMSC on CD4+IFN-γ+ T lymphocytes, but the TGF-β1 inhibitor had no similar effect. This study found that the regulation effects of OMSC improved neurological function and reduced IFN-γ secretion in EAE mice. The downregulation of IFN-γ by OMSC may be related to the inhibition of CD4+IFN-γ+ T cells, and the COX pathway may be involved in this progress.

In this study, although the role of the COX pathway in the regulation of human CD4+IFN-γ+ T cells by OMSC was explored, the effects of COX-1 and COX-2 inhibitors and their downstream pathway were not further studied. In addition, the immunomodulatory effect of OMSC on CD4+IFN-γ+ T lymphocytes in the spinal lesions and spleen of EAE mice, as well as the immunomodulatory effect of OMSC on human lymphocytes and the related mechanisms, need to be further studied, which is also an essential way to achieve clinical application of OMSC.

## Conclusion

In conclusion, we have demonstrated that OMSC transplantation delayed the onset and promoted neural recovery in the EAE model. OMSC modulate CD4+IFN-γ+ T cells, and the COX pathway is involved in the immunomodulatory progress. Thus, OMSC are a potential candidate for the treatment of neurological autoimmune disease.

## Methods

### Acquisition of Human OMSC

The olfactory mucosa of healthy donors was obtained through a nasal endoscope under local infiltration anesthesia with 2% lidocaine. The olfactory mucosa was cut with sterile instruments, digested with collagenase IV at 37°C for 1 h, and then passed through a 70-μm cell sieve (BD, CA, United States). After centrifugation at 4°C, 300 g, the cell suspension was resuspended and transferred into a culture bottle. The low-glucose DMEM (Gibco, Grand Island, NY, United States) was prepared with 10% FBS (Gibco, Grand Island, NY, United States), 100 U/ml penicillin (Gibco, Grand Island, NY, United States), and 0.1 g/L streptomycin (Gibco, Grand Island, NY, United States) (Ge et al., 2016). At the fifth passage, surface molecular identification and multi-differentiation staining and cell suspension preparation were performed.

### Acquisition of Human UMSC

The umbilical cord tissue was taken from puerpera and washed with normal saline three times, with blood vessels removed. The umbilical cord tissue was cut and soaked in a solution containing 100 U/ml penicillin (Gibco, Grand Island, NY, United States) and 0.1 g/L streptomycin (Gibco, Grand Island, NY, United States). After digesting with collagenase I and hyaluronidase at 37°C for 5 h, centrifuging at 4°C, 300 g, and passing through a 70-μm cell sieve, the cells were resuspended and transferred into a culture bottle (Corning, NY, United States). The low-glucose DMEM (Gibco, Grand Island, NY, United States) was prepared with 10% FBS (Gibco, Grand Island, NY, United States), 100 U/ml penicillin (Gibco, Grand Island, NY, United States), and 0.1 g/L streptomycin (Gibco, Grand Island, NY, United States). At the fifth passage, surface molecular identification and multi-differentiation staining and cell suspension preparation were performed.

### Acquisition of Peripheral Blood Lymphocytes From Healthy Donors

After peripheral blood was acquired, polysucrose solution (Serumwerk Bernburg AG, Alere Technologies, Oslo, Norway) and density gradient centrifugation were used to separate peripheral blood lymphocytes. After purification using red blood cell lysis buffer (Solarbio, Beijing, China), the peripheral blood lymphocytes were resuspended and counted, and cultured in a CO_2_ incubator at 37°C.

### Construction of the Co-Culture System

OMSC (or UMSC) were transferred into a 24-well plate (Thermo Fisher Scientific, Waltham, MA, United States) at a density of 1×10^5^ cells/well; CD3+ lymphocytes were purified using CD3 MicroBeads (Catalog # 130-050-101, Miltenyi Biotec, Bergisch Gladbach, Germany) and transferred into a 24-well plate at a density of 5×10^5^ cells/well. The total culture volume was 500 μl. For the *in vitro* co-culture study, each well was stimulated with 12.5 μg MOG_35-55_. For the *in vitro* mechanism study, each well was stimulated with 500 ng anti-CD28 (BD, CA, United States) and 100 ng anti-CD3 (BD, CA, United States). For the COX inhibitor group, indomethacin (1 uM, Sigma-Aldrich, St. Louis, MO, United States) was added to the culture system, and for the TGF-β inhibitor group, anti-TGF-β (1 ug/ml) antibody was added to the culture system. At the last 6 h, the cells were stimulated with PMA (50 ng/ml) and ionomycin (500 ng/ml). Brefeldin A (BFA, 10 μg/ml) was used to inhibit the secretion of cytokines. Each experiment was repeated three times (*n* = 3).

### Detection of Surface Markers and Intracellular Factors of Human Lymphocytes

The cells were collected and washed with PBS (Gibco, Grand Island, NY, United States) for staining. The cells were resuspended with 100 μl staining buffer, stained with APC-CD8 (BD, CA, United States) at 4°C for 30 min, fixed with 4% paraformaldehyde (Boster Biological Technology, Wuhan, China), stained with PE-Cy7-IFN-γ (BD, CA, United States), permeabilized (Invitrogen, CA, United States) at 4°C for 30 min, and washed and resuspended with PBS.

### Surface Molecular Identification

The fifth passage of UMSC were digested with 0.25% trypsin (Gibco, Grand Island, NY, United States), and OMSC were digested with 0.125% trypsin at 37°C. The concentration of the cell suspension was 1×10^6^/ ml; it was filtered by a 70-μm sieve (BD, CA, United States) and transferred to flow tubes. UMSC and OMSC were labeled with PE-CD29 (BD, CA, United States), PE-CD90 (BD, CA, United States), PE-Cy7-CD34 (BD, CA, United States), PE-Cy7-CD45 (BD, CA, United States), APC-CD44 (BD, CA, United States), APC-CD166 (BD, CA, United States), FITC-CD73 (BD, CA, United States), and FITC-CD105 (BD, CA, United States) at room temperature for 30 min. The surface molecules were detected by flow cytometry.

### Alizarin Red S Staining

The fifth passage of UMSC (or OMSC) were transferred into 6-well plates (Thermo Fisher Scientific, Waltham, MA, United States) at the density of 3×10^5^/well. After 80% density, the old culture medium was removed, with a culture medium for the induction of osteoblasts by a previous study (Lei et al., 2013). The induction culture was carried out in the incubator with 5% CO_2_ under 37°C, and half of the medium was changed every 2 days. At the 21st day of culture, the medium was sucked out, and the cells were washed with PBS buffer three times, fixed with 4% paraformaldehyde (Boster Biological Technology, Wuhan, China) for 15 min, stained with 0.1% Alizarin Red S (Rolex-Bio, Guangzhou, China) for 10 min, and washed with PBS three times.

### Oil Red O Staining

The fifth passage of UMSC (or OMSC) were transferred into 6-well plates at the density of 3×10^5^/well. After 80% density, the culture medium was removed, with a culture medium for the induction of lipoblasts by a previous study (Lei et al., 2013). After induction with medium A for 3 days, fat induction medium B was used for 1 day. After circulation for 21 days, the medium was sucked out, and the cells were washed gently with PBS once and fixed with 4% paraformaldehyde for 15 min. After staining the cells using 0.5% Oil Red O (Sigma-Aldrich, St. Louis, MO, United States) for 10 min, they were washed carefully with isopropanol (Guangzhou chemical reagent factory, Guangzhou, China), gently rinsed with PBS three times, and then observed and photographed under a microscope.

### Animals

C57BL/6 female mice were provided by Charles River, Ltd. All animal experiments, breeding, and care were performed according to Animal Experiment Center Guidelines and approved by the Animal Ethics Committee of Sun-Yat sen University.

### EAE Model Induction

EAE was induced in 6 to 8-week-old female C57BL/6 mice. The mice were anesthetized using 10 g/L pentobarbital sodium (Sigma-Aldrich, St. Louis, MO, United States) at a dose of 50 mg/kg. The pedal reflex and tail pinching reflex were observed to ensure that the mice were anesthetized. The skin was prepared on the back of the mice, and the mice were labeled with ear markers. An emulsion containing 1 mg/ml myelin oligodendrocyte glycoprotein (MOG) _35–55_ (Sigma-Aldrich, St. Louis, MO, United States) together with complete Freund adjuvant (CFA, Sigma-Aldrich, St. Louis, MO, United States) with 4 mg/ml of *M. tuberculosis* H37R (BD, CA, United States) was injected subcutaneously on both sides of the back of the mice. In addition, 500 ng of pertussis toxin (List Biological Laboratories, New Delhi, INDIA) was injected intraperitoneally on days 0 and 2. All mice were randomly allocated to the OMSC treatment (*n* = 6), UMSC treatment (*n* = 6), and PBS groups (*n* = 7).

The mice were scored daily for neurological function evaluation according to the 6-point EAE scale as mentioned in a previous study (Ben-Zwi et al., 2019): 0, asymptomatic; 1, partial loss of tail tonicity; 2, tail paralysis; 3, hind limb weakness; 4, hind limb paralysis; 5, 4-limb paralysis; and 6, death.

### Cell Therapy

On the 9^th^ day after immunization, the third to fifth passage of OMSC and UMSC were digested, filtered, and counted. According to the counting results, an appropriate number of cells were resuspended to equal concentration (1×10^6^/200 μl) of the cell suspension. The mice were fixed on the tail vein injection instrument, and the injection points were treated with 75% alcohol (Guangzhou Chemical Reagent Factory, Guangzhou, China). A 1-ml insulin needle (BD, CA, United States) was used to inject OMSC, UMSC cell suspension (1×10^6^/mouse), or PBS. The mice in OMSC treatment, UMSC treatment, and control groups were injected with cells or PBS *via* the tail vein.

### Histopathology

After the mice were sacrificed, PBS and 4% paraformaldehyde were used in turns to perfuse, and spinal cords were carefully removed and fixed with 4% paraformaldehyde for 24 h, and then dehydrated using 75–95% ethanol. And the tissue was cleared with xylene, and a paraffin-embedded tissue block was made and was cut into paraffin sections.

Hematoxylin and eosin (HE) staining: The paraffin sections were put into ethanol and xylene for gradient dehydration. The hematoxylin staining solution was added to dye for 5 min and then washed with running tap water, followed by eosin dye staining for 5 min. After dehydrating, the sections were sealed with a sealing agent. Inflammation was scored as follows: 0, no inflammatory cells; 1, a few scattered inflammatory cells; 2, organization of inflammatory infiltrates around blood vessels; and 3, extensive perivascular cuffing with extension into the adjacent parenchyma, or parenchymal infiltration without obvious cuffing.

Luxol fast blue (LFB) staining: The paraffin sections were put into ethanol and xylene for gradient dehydration. Myelin staining solutions A and B were preheated, and the slices were put into dye A, dyed for 3 h, and then taken out and washed with water. After being immersed in staining solution B, the differentiation was terminated. After dehydrating, the sections were sealed with a sealing agent. Demyelination was scored as follows: 0, none; 1, rare foci; 2, a few areas of demyelination; and 3, large (confluent) areas of demyelination (Calida et al., 2001).

### Cytometric Bead Array

After anesthetization, the mice were sacrificed and peripheral blood was collected through the inner canthus vein with a capillary collecting vessel. After centrifugation at 4°C for 10 min, the upper serum was transferred to a new EP tube. The standard sample was diluted, and the standard curve was made according to the CBA kit’s (BD, CA, United States) operation instructions. The concentration of IFN-γ, TNF-α, IL-17, IL-6, and IL-10 was detected by flow cytometry.

### Analysis of the Spleen Lymphocyte of EAE Mice

On the day of EAE onset, the mice were anesthetized and sacrificed. The spleen of mice of OMSC treatment and control groups was observed and ground (*n* = 3). Red blood cell lysis buffer was used to remove red blood cells. After washing and centrifugation, the lymphocytes were resuspended in 1640 medium (Gibco, Grand Island, NY, United States) and counted by using the cell counting plate. Purified lymphocytes were transferred into 24-well plates at a density of 1×10^6^ cells/well. The volume of the culture system was 500 μl. Five microliters of leucocyte activation cocktail (BD, CA, United States) was added to each well to stimulate the cells for 6 h. After washing with PBS twice, the cells were resuspended in 100 μl PBS. After staining with FITC-CD3 (BioLegend, San Diego, CA, United States) and APC-CD4 (BioLegend, San Diego, CA, United States) at 4°C for 30 min, the lymphocytes were fixed with 4% PFA at room temperature for 15 min, then permeabilized, and stained with PE-Cy7-TNF-α (BioLegend, San Diego, CA, United States) and PE-IFN-γ (BioLegend, San Diego, CA, United States) at 4°C for 30 min. After washing with PBS, the lymphocytes were resuspended with PBS. The proportions of T lymphocyte subsets were detected by flow cytometry. Each experiment was repeated three times (*n* = 3).

### Statistical Analysis

GraphPad Prism 8 and SPSS 20.0 were used for graphing and statistical analysis. All data were expressed as mean ± SEM. The Kruskal–Wallis *H* test was used to evaluate the neurological function score of mice among three groups; one-way ANOVA was used for detecting differences in the mean values of the three groups, and the *LSD-t* test was used for pairwise comparison. A *p* value < 0.05 was considered statistically significant.

## Data Availability

The original contributions presented in the study are included in the article further inquiries can be directed to the corresponding authors.

## Abbreviations

CBA, cytometric bead array; CD29/34/44/45/73/90/105/166, cluster of differentiation 29/34/44/45/73/90/105/166; CNS, central nervous system; COX, cyclooxygenase; DMT, disease-modifying therapy; EAE, experimental autoimmune encephalomyelitis; IFN-γ, interferon γ; IL-6/10, interleukin-6/10; LFB, luxol fast blue; MOG, myelin oligodendrocyte glycoprotein; MSC, mesenchymal stem cells; OMSC, human olfactory mucosa–derived mesenchymal stem cells; PG, prostaglandin; TGF-β1, transforming growth factor-β1; TNF-α, tumor necrosis factor α; UMSC, human umbilical cord–derived mesenchymal stem cells.
